# Antibacterial Effect of a 4x Cu-TiO_2_ Coating Simulating Acute Periprosthetic Infection—An Animal Model

**DOI:** 10.3390/molecules22071042

**Published:** 2017-06-23

**Authors:** Andreas Mauerer, Stefanie Stenglein, Stefan Schulz-Drost, Christoph Schoerner, Dominic Taylor, Sebastian Krinner, Frank Heidenau, Werner Adler, Raimund Forst

**Affiliations:** 1 Department of Trauma and Orthopaedic Surgery, St. Theresa-Hospital Nuremberg, Mommsenstr. 24, 90491 Nuremberg, Germany; dominictaylor2000@yahoo.com; 2 Biomechanics Laboratory-UO Lab, Department of Trauma and Orthopedic Surgery, University Hospital Erlangen Krankenhausstr. 12, 91054 Erlangen, Germany; 3 Abteilung für Unfall-, Hand-, und Orthopädische Chirurgie, Sana Kliniken Solln Sendling, Plinganserstraße 122, 81369 München, Germany; stefanie.stenglein@gmx.de; 4 Department of Trauma and Orthopedic Surgery, University Hospital Erlangen, Krankenhausstr. 12, 91054 Erlangen, Germany; stefan.schulz-drost@uk-erlangen.de (S.S.-D.); sebastian.krinner@uk-erlangen.de (S.K.); 5 Institute of Microbiology, Clinical Microbiology, Immunology and Hygiene, Universitätsklinikum Erlangen, Wasserturmstraße 35, 91054 Erlangen, Germany; christoph.schoerner@uk-erlangen.de; 6 BioCer Entwicklungs GmbH, Ludwig-Thoma-Straße 36, 95447 Bayreuth, Germany; frank.heidenau@biocer-gmbh.de; 7 Department of Medical Informatics, Biometry and Epidemiology, Friedrich-Alexander-University Erlangen-Nuremberg, Waldstr.6, 91054 Erlangen, Germany; werner.adler@imbe.med.uni-erlangen.de; 8 Department of Orthopaedic Surgery, Friedrich-Alexander-University Erlangen-Nuremberg, Rathsbergerstr. 54, 91054 Erlangen, Germany; mail@rforst.de

**Keywords:** antibacterial Cu-TiO_2_ coating, acute implant-associated infection, animal model, total knee arthroplasty, copper-ions

## Abstract

The purpose of our study was to investigate the antibacterial effect of a spacer (Ti6Al4V) coated with 4x Cu-TiO_2_ in an animal model simulating an acute periprosthetic infection by *Staphylococcus aureus*. Ti6Al4 bolts contaminated with *Staphylococcus aureus* were implanted into the femoral condyle of rabbits (n = 36) divided into 3 groups. After one week in group 1 (control) the bolts were removed without any replacement. In group2 Ti6Al4V bolts with a 4x Cu-TiO_2_ coating and in group 3 beads of a gentamicin-PMMA chain were imbedded into the borehole. Microbiological investigation was performed at the primary surgery, at the revision surgery and after scarification of the rabbits 3 weeks after the first surgery. Blood tests were conducted weekly. The initial overall infection rate was 88.9%. In group 2 and 3 a significant decrease of the infection rate was shown in contrast to the control group. The C-reactive protein (CRP) levels declined one week after the first surgery except in the control group where the CRP level even increased. This is the first in vivo study that demonstrated the antibacterial effects of a fourfold Cu-TiO_2_ coating. For the future, the coating investigated could be a promising option in the treatment of implant-associated infections.

## 1. Introduction

In most industrialized countries, the number of joint replacements has significantly increased during previous years. Recent studies published by the Organisation for Economic Co-operation and Development (OECD) prove that the number of hip replacements in its member states has increased on average by 35%, whereas the rate of knee replacements actually doubled in the same time frame between 2000 and 2013 [1]. Although the history of joint replacement is generally recognized as a success story, periprosthetic infections after knee replacements still occur with an incidence of approximately 1.6% within the first 2 years with a predicted overall cost for the treatment of periprosthetic infections coming to more than $50,000 per case [2,3]. Although there is still not one clear evidence based strategy for the effective treatment of periprosthetic infections, most orthopedic surgeons agree to remove the implant immediately after infection [4,5].

Staphylococci, such as *Staphylococcus aureus* and coagulase-negative species, cause most of the periprosthetic infections [6,7,8]. In scientific literature, coatings loaded with antibiotics, or with non-antibiotic organic antimicrobial agents and adhesion resistant coatings are frequently discussed [9]. Previous studies by Heidenau et al. [10] could show that 4x Cu-TiO_2_ coated titanium alloys (Ti6Al4V), are associated with both a great antibacterial effect and an excellent tissue compatibility. In an animal model it could be demonstrated that the above-mentioned coating is an active coating whose antibacterial ingredients are released over a duration of 4 weeks [11].

The purpose of the following study was to investigate the antibacterial effect of a spacer (Ti6Al4V) coated with 4x Cu-TiO_2_ in an animal model simulating an acute periprosthetic infection by *Staphylococcus aureus* after total knee arthroplasty (TKA).

The results are compared firstly to those of an implanted gentamicin-polymethylmethacrylate (PMMA) chain and secondly to the simple removal of the infected implant.

## 2. Results

### 2.1. Microbiological Results

During the first surgery, bone material from the borehole was examined microbiologically. All samples were sterile. This means that before the inoculation with *Staphylococcus aureus*, no infection was present within the laboratory animals.

After inoculation with *Staphylococcus aureus*, a total of 32 out of the 36 rabbits were infected which corresponds with an overall infection rate of 88.9%.

Our microbiological findings were classified as an infection when the presence of *Staphylococcus aureus* was detected.

In group 1 (n = 12) two rabbits died because of sepsis before the first revision and consequently had to be excluded from the study ahead of schedule.

The infection rate of the remaining animals of group 1 (n = 10) before revision and after euthanasia was 80%. Thus, there was no change in the infection rate seen on removal the infected implant (*p* = 1).

In group 2 (n = 12) one laboratory animal died before euthanasia and was not considered in our research study. One week after implantation of the titanium-aluminium screw over 90% of the observed rabbits were infected. Two weeks after the insertion of the copper titanium screws (at the time of euthanasia), the infection rate fell significantly to 41.7% (*p* = 0.01).

**Figure 1:**
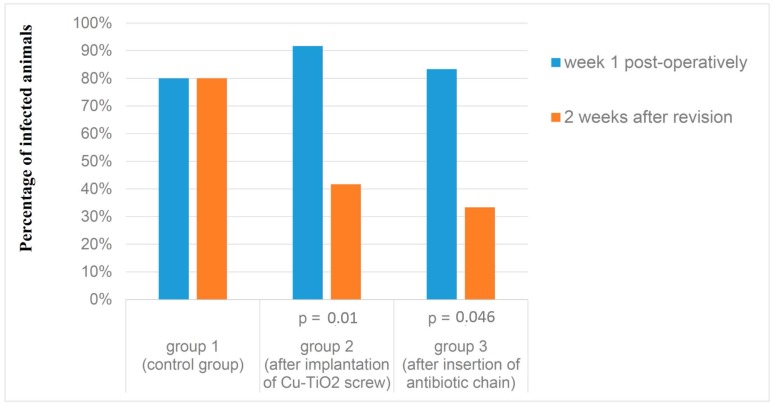
Comparison of the “rate of infection” at the time of revision and post mortem.

In group 3 (n = 12) one animal died before euthanasia and was consequently excluded from our study. In this group 83.3% of the laboratory animals were infected after primary surgery. Two weeks after removal of the aluminum-titanium screws and implantation of the antibiotic chain, only 33.3% of the animals were infected. This decrease was statistically significant (*p* = 0.046) (Figure 1).

The Mann-Whitney-U-Test shows that there is a significant difference between group 1 and group 2 (*p* = 0.005), but not between group 1 and group 3 (*p* = 0.08). There was also no significant difference between group 2 and group 3 (*p* = 0.35).

### 2.2. Blood Tests

The baseline serum Cu concentration was similar in each of the groups.

One week after implantation of the titanium-aluminium implant and inoculation of the bacteria, the maximum peak of Cu concentration could be observed in each group, though the maximal peak in group 3 was significantly higher than in group 2 and group 1 (*p* < 0.001).

**Figure 2:**
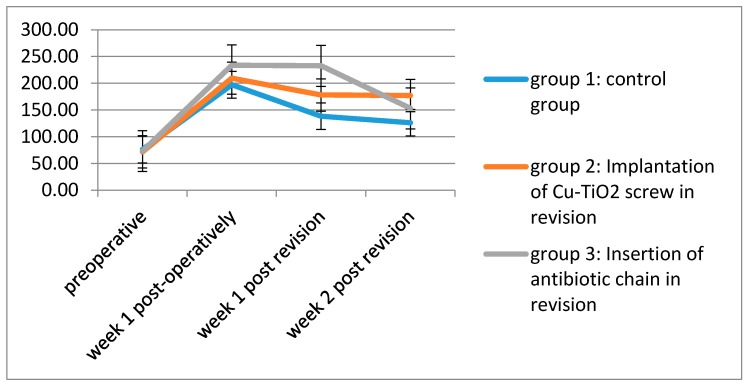
Mean copper serum levels (μg/dL) of the three groups over the examination period.

After reaching the peak value, the Cu level in group 1 decreased fast and continually, whereas in group 2 the Cu concentration hardly changed. In group 3 the Cu concentration had a slow decrease followed by a rapid fall to 153.04 µg/dL two weeks after revision (Figure 2, Table 1).

**Figure 3:**
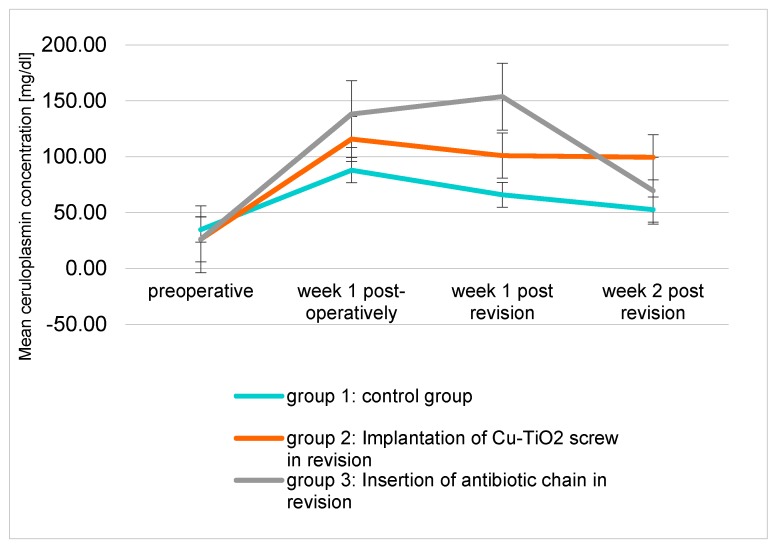
Mean ceruloplasmin concentration (mg/dL) of the three groups over the examination period.

The serum concentration of ceruloplasmin in group 1 and group 2 reached its maximum peak after the first week and then decreased steadily over time. In group 3, the maximum of ceruloplasmin was reached one week after revision and then dropped rapidly to 69.53 µg/dL two weeks after the revision (Figure 3).

The CRP serum levels showed the peak value one week after the first surgery in each of the three groups. In group 2 and group 3 the maxima were significantly higher than in group 1.

**Figure 4:**
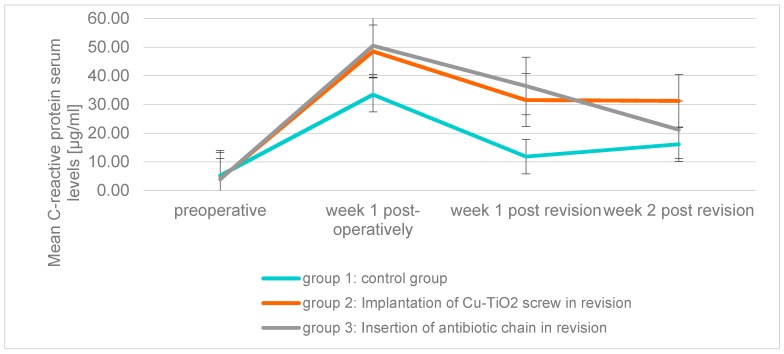
Mean C-reactive protein serum levels (μg/mL) of the three groups over the examination period.

In each group, the decrease in CRP levels was very dynamic one week after primary operation. Whereas in group 3 a further decline of CRP level could be observed from the first to the second week after revision, the value stagnated in group 2 or even slightly increased in group 1 (*p* = 0.412) (Figure 4).

In comparison to the CRP values, the leukocytes showed a delayed increase. The maximum value of leukocytes in each group was attained merely one week after the revision surgery.

**Figure 5:**
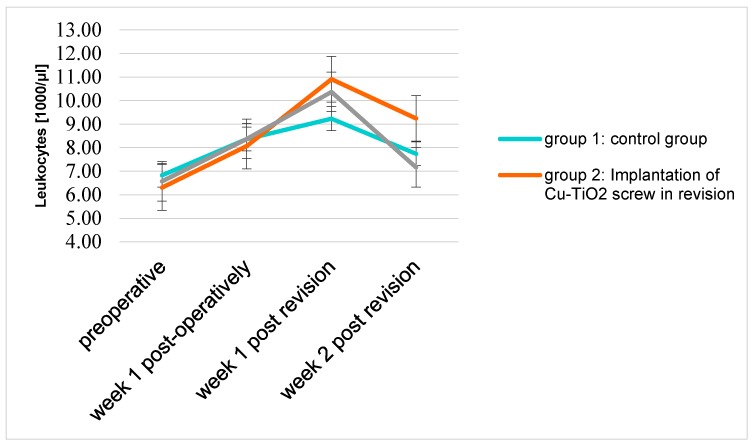
Mean leukocyte count (1000/μL) of the three groups over the examination period.

Although there was no significant difference between the group 1 and 2 (*p* = 0.2), group 2 showed the highest peak of leukocyte-increase with 10. 9 (1000/μL) in the first week after revision (*p* = 0.2) (Figure 5).

**Figure 6:**
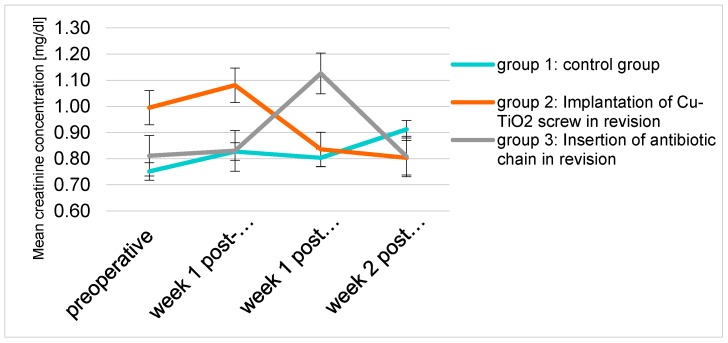
Mean creatinine concentration (mg/dL) of the three groups over the examination period.

In all 3 groups the serum creatinine, as an indicator of renal health, stayed clearly within the normal range (Figure 6).

**Figure 7:**
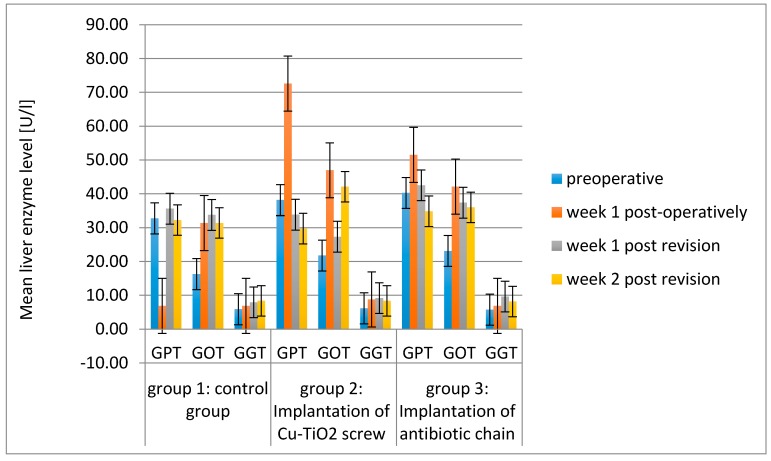
Mean Liver Enzyme Level (gamma-glutamyl transferase (GGT), glutamic-pyruvate transaminase (GPT) and glutamic-oxaloacetate transaminase (GOT)) (U/l) of the three groups over the examination period

The values for gamma-glutamyl transferase (GGT), glutamic-pyruvate transaminase (GPT) and glutamic-oxaloacetate transaminase (GOT), representing the liver enzymes, were, with the exception of GPT in group 2 after the implantation of the copper-titanium screw, at every point in time and in each group within the standard range (Figure 7) [12].

## 3. Discussion

The present study analyzed whether there is any difference in the treatment outcomes of implant-associated infections with reference to microbiological examinations, serological infection parameters, copper serum levels, as well as liver and kidney values, between: solely removing the infected implant (group 1), its replacement with an antibacterial coated implant (4x Cu-TiO_2_) (group 2), or the insertion of an antibiotic chain together with removal of the bolt (group 3).

To standardize the comparison of the different groups mentioned above, we decided in advance that no surgical debridement should be performed, despite the fact that this is considered to be good clinical practice in the treatment of implant associated infections [13].

In total 32 out of 36 animals were infected, representing a total infection rate of 88.9%. Four laboratory animals died in the course of the study prematurely due to sepsis.

In comparison to other existing animal models of early postoperative infection, we were able to achieve a highly reproducible rate of infection (Figure 1) [14].

Our data suggest that the simple removal of the screw without replacement did not result in an improvement of infection rates at all. Our results are partly in line with other studies [15,16,17,18,19,20,21].

Even if good results are reported after first stage revision procedures in cases of implant related infection, the insertion of spacers before implanting a new prosthesis seems to bring considerable benefits [15,16,17,18,19,20,21]. This might be explained by the fact, that despite the most careful debridement, of course not all pathological bacteria can be eliminated.

Using an antibacterial spacer is supposed to further reduce germination and at least a second debridement would follow prior to reimplantation of the definitive implant.

As one of the main findings the study demonstrated, that the rate of infection among the animals could be decreased significantly from 90% to 41.7% after 2 weeks by the application of a spacer coated with 4x Cu-TiO_2_ (group2) (Figure 1).

These results also confirm previous in vitro data of Heidenau et al. [10] proving that copper ions (4x Cu-TiO_2_) applied on a Ti6Al4V were highly effective against bacteria combined with proven tissue tolerability.

Nevertheless, this is the first time that a significant antibacterial effect of the tested 4x Cu-TiO_2_ coating could be shown in an in vivo model simulating an early periprosthetic infection after total knee arthroplasty.

The chronological sequence of the release of the antibacterial copper ions of the active coating with a maximum peak one week after implantation of the antibacterial coating is similar as described before in a similar animal model without infection [11]. This could explain the antibacterial effect within the first two weeks after the implantation of the antibacterial implant.

In group 3 three PMMA beads containing gentamicin were inserted into the respective borehole, which also corresponds to good clinical practice. In this group the infection rate of formerly 89% could be significantly reduced by insertion of a gentamicin chain to 33.3% (Figure 1).

Although these numbers could lead to the conclusion that the use of antibiotic chains reached even better success rates than the insertion of copper coated implants, the Mann-Whitney-U-Test showed that there was no a significant difference between group 2 and group 3 (*p* = 0.35).

This could be explained statistically by the one weak point of our study, the relatively small size of the groups. To achieve more detailed results, larger group sizes would be necessary.

On assessment of the copper and ceruloplasmin serum levels in each group, it was noticeable that each group reached its peak value one week after the first surgery, at a time when the infected TiAl implant was still in situ.

This can be explained by the fact that ceruloplasmin is the major copper-carrying protein in the blood.

Furthermore ceruloplasmin is synthesized and secreted by hepatocytes, accounts for 95% of total copper in circulation [22,23]. Ceruloplasmin is the key source of ferroxidase I transit, which reduces free radicals and is also an acute phase protein, raised during infections [9,24,25].

After revision surgery, the copper and ceruloplasmin serum levels in group 1 and group 3 decreased. In group 2 the copper serum level stayed almost even in week 1 and 2 after the revision surgery (*p* = 0.01). This is partly in line with former studies where it could be shown that this coating is an active coating, releasing copper ions over a period of several weeks [11].

Regularly monitored blood tests validate that GGT ranged within normal values in each group, whilst the GOT was significantly elevated above the norm in all measurements (except for the baseline value) in groups 2 and 3 (Table 1, Figure 7). This could be related to the fact that GOT is not just a liver-specific enzyme but also occurs in striated muscle and therefore increases in skeletal muscle trauma, as occurred during the intramuscular injection for the induction of the anaesthesia and during surgery [26].

Gopinath et al. [27] demonstrated significant morphological modifications in the kidney due to chronic copper poisoning. To monitor for this eventuality, and to detect any possible systemic effects of copper, weekly creatinine samples were taken. During the term of validity all creatinine average values remained in the normal range (Table 1, Figure 6) [12].

In spite of the restricted number of laboratory animals in the study (n = 36), the reported data substantiated the effectiveness of the antibacterial coating investigated. Nevertheless, a larger sample size may strengthen the results even more. However, in our case this was not possible due to ethical concerns.

## 4. Materials and Methods

### 4.1. Animals

All procedures involving rabbits were performed according to the contract as approved by the government of the administrative district authorized for the first author’s institution.

Since there are no analogous studies, a study with 36 New Zealand White (NZW) rabbits (n = 36) was set up. They were separated into three groups (group 1–3) of twelve animals each (n = 12), to obtain meaningful results by an appropriate group size.

The mean rabbit weight was 4.228 g (SD: 273.364).

The rabbits were allowed to acclimatize for 7 days to the new environment before the first operation took place. All animals were accommodated in separate cages in a climate-controlled facility with open access to antibiotic-free commercial pellets (Rabbit Maintenance, ssniff^®^, Soest, Germany) and water. A veterinarian examined the health status and suitability of each rabbit before the first surgery.

### 4.2. Design of the Implants

**Figure 8:**
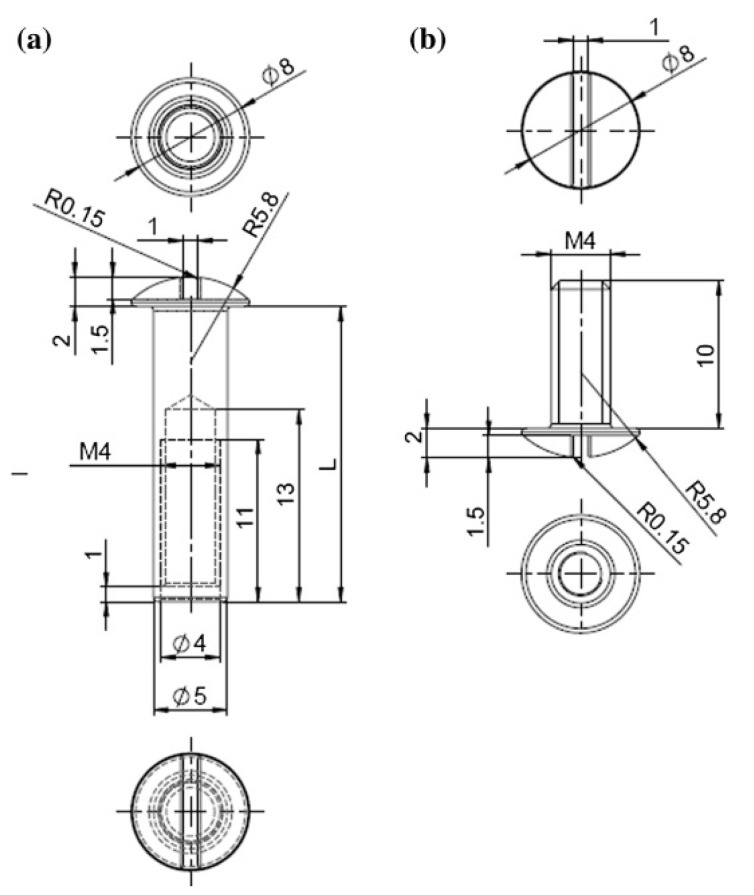
Design of bolt with internal screw thread (**a**) and lock screw (**b**). All dimensions shown in mm, L = length, M4 = metric internal screw thread (ISO standards), R = radius.

**Figure 9:**
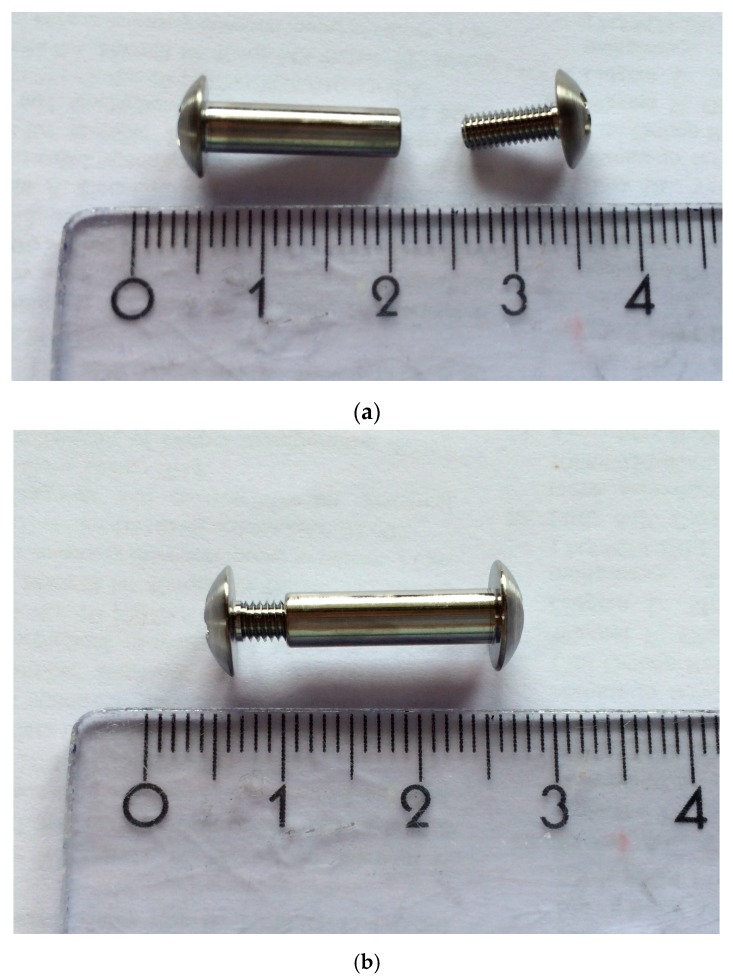
(**a**) 5mm diameter Titanium alloy (Ti6Al4V) bolts (medrasys GmbH, medical technologies, Pressig, Germany) and 4 mm metric internal screw thread (ISO standards); (**b**) Example of an original locked bolt and screw.

We used custom made 5 mm diameter Titanium alloy (Ti6Al4V) bolts (medrasys GmbH, medical technologies, Pressig, Germany) with a flat surface and a 4 mm metric internal screw thread (ISO standards) for a lock screw (Figure 8a,b and Figure 9a,b). The screw head and the head of the locking screw were suitable for standard screwdrivers.

In group 2 identically designed bolts were coated with four layers of Cu-TiO_2_.

### 4.3. Preparation of the Copper Modified TiO_2_-Coatings

The Ti6Al4V bolts were modified at the surface with crystalline copper-containing titanium oxide layers, as described before [10,28]. Titanium oxide coatings in general have been reported to show high stability paired with excellent biocompatibility under physiological conditions [29].

Prior to the coating process, the implants were cleaned with water and rinsing agent, sonicated in dry ethanol for 2 min to remove organic residuals and finally dried with cyclohexane and acetone.

Copper ions were included into the titanium sol by cold saturation with Cu-(II)-acetate monohydrate (Merck, Darmstadt, Germany). The specimens were then coated by a dip coating process with a speed of withdrawal of 1.5 mm/s and an immersion time of 20 s in the sole.

The formation of the copper containing titanium oxide layer on the implants in combination with hydrolysis of the organic matrix by humidity was done under controlled atmospheric conditions (40% rel. humidity, 25 °C, 1 h). The calcination to the inorganic titanium-oxide coating was accomplished in a furnace at 500 °C. To achieve the final implants, the dip coating process was performed four times.

### 4.4. Antibiotic Chain

In group 3 a modified Septopal^®^ Minichain (Biomet, Berlin, Germany) was inserted. This minichain contains 3 oval shaped pellets (diameter 3 × 5 mm) arrayed on a polyfile steel wire and each of them contains 2.8 g gentamicinsulphate (≙ 1.7 gentamicin base).

### 4.5. Surgical Procedure

All surgical procedures were performed under anaesthesia by the same orthopaedic surgeon and with the supervision of a veterinarian.

Anaesthesia was performed as described before [11].

Each rabbit had a different set of instruments for each surgery and every operation was accomplished under sterile conditions. Only the right hind leg of each animal was operated. Initially the thighs and hind legs of the respective leg were shaved and the area concerned was disinfected with an antiseptic fluid (Skinsept^®^ F, Ecolab GmbH, Düsseldorf, Germany).

Sterile adhesive surgical drapes were wrapped around the right hind leg. Then a 2 cm skin incision on the lateral side of the knee was carried out and afterwards an arthrotomy was performed. Thereafter, the lateral femoral condyle and the approach of the lateral collateral ligament (LCL) were uncovered and a 5.0 mm bicortical drill hole was performed. Thus, an intra-articular defect in the non-weight, non-articulated aspect of the lateral femoral condyle was accomplished. The bone material which was released from the drill hole was kept for microbiological examination. It could be demonstrated that all samples were taken under sterile conditions.

Before application of the titanium-aluminum screw, 10^5^ colony forming units (CFU) of *Staphylococcus aureus* were introduced into the drill hole.

On the opposite side, a small skin incision was made and a screw with an internal thread was inserted into the borehole and locked from the other side. In each group uncoated Ti6Al4V bolts were implanted, as described before (Figure 8a,b and Figure 9a,b). Subsequently to hemostasis, the joint capsule was sealed with a locked running non-absorbable 4–0 monofilament nylon. Finally, the open skin was readapted with an absorbable 3–0 suture.

After one week the revision surgery was accomplished in each group in which the same surgical approach was used, and the titanium-aluminum screw was removed.

In group 1 the screw was removed without reimplanting any implant.

In group 2 Ti6Al4V bolts with four TiO_2_-coatings containing Cu^2+^-ions (4x Cu-TiO_2_) were implanted and subsequently were removed two weeks after the euthanasia.

In group 3 three beads of a gentamicinsulphate minichain (Septopal®Minichain, Biomet, Berlin, Germany) were inserted after removal of the screw and were left inside the borehole for the following two weeks until euthanasia.

The removed implant was preserved for further microbiological examinations and placed into a sterile container used for the preservation of microbiological specimens in daily clinical practice.

After surgery each rabbit was observed until full recovery from anaesthesia. Postoperative analgesia was achieved by subcutaneous injection of 0.01–0.05 mg/kg buprenorphine (Buprenovet^®^, Bayer Vital, Leverkusen, Germany).

### 4.6. Blood Samples

Altogether four blood samples from an ear vessel were taken over a period of four weeks.

The first preoperatively, the second 1 week after primary implantation just before revision surgery, the third in the first week after revision surgery and the fourth just before sacrificing the animals.

The following parameters were determined: level of Cu (µg/dL), C-reactive protein (CRP, µg/mL), leukocytes (100/µL), ceruloplasmin (CP, mg/dL), gamma-glutamyl transferase (GGT, U/L), glutamic-pyruvate transaminase (GPT, ALT, U/L), glutamic-oxaloacetate transaminase (GOT, AST, U/L), and serum creatinine (mg/dL). Those parameters were selected to monitor possible inflammation, organ function and possible systemic effects of copper.

### 4.7. Euthanasia

After the fourth blood test, the rabbits were euthanized two weeks after revision surgery according to our protocol and the directive of the European Union by intravenous injection of 200 mg/kg pentobarbital (Euthadorm Bayer Vital, Leverkusen, Germany).

### 4.8. Microbiology

During the first operation, one Milliliter of 10^5^ CFU *Staphylococcus aureus* ATCC 29213/DSZM 2569 was injected into the borehole before insertion of the Titanium alloy bolts (Ti6AlV). A new bacterial suspension was manufactured before every procedure. First of all, the germ suspension was brewed overnight in a Tryptic soy broth and then centrifuged out. It was then resuspended in physiological saline solution. Photometry was used to calibrate the solution to a concentration of McFarland 0.5, which corresponds to circa 10^8^ CFU/mL.

To achieve the final concentration of 10^5^ CFU/mL, a further dilution with 0.9% NaCl was carried out. This was verified by growing a sample on Muller-Hinton-Agar.

The microbiological examinations of the bony material from the borehole (from the first procedure), the explanted Titanium-Aluminium bolts, the Copper-Titanium bolts and the Septopal micro chains were carried out as follows:

The sterility of the primary bony material samples was tested on solid medium (Columbia-blood agar, Chocolate blood agar and Vitamin K agar) and liquid medium (Tryptic soy broth and Thioglycolate broth).

The explanted bolts and mini chains were rolled onto Columbia blood agar. Afterwards, for detection purposes, a small sample of the germs was placed into Tryptic soy broth. The tally of the germ growth was semi-quantitatively recorded.

The cultured germs were identified by use of a mass spectrometer with matrix-assisted laser desorption/ionization (MALDI-TOF).

The presence of *Staphylococcus aureus* on the explanted bolts and/or on the Septopal mini chains was classified as an implant associated infection or respectively as the further existence of an infection.

### 4.9. Statistics

Mixed-effects regression models were calculated with time and group as independent variables and blood parameters as dependent variables. *p* values < 0.05 were evaluated as statistically significant.

To distinguish differences between the three groups for a specific time, Mann-Whitney-U-tests were used and the p-values were adjusted for multiple tests with the Bonferroni method.

All statistical analysis was carried out by using the statistical programming language R [30] and the software SPSS (IBM Corp. Released 2012. IBM SPSS Statistics for Windows, Version 21.0. IBM Corp, Armonk, NY, USA).

## 5. Conclusions

The results of this paper lead to the conclusion that our animal model is suitable as an animal model for simulating early implant-associated infections in total joint replacement.

Moreover, this was the first in vivo study that could show antibacterial effects of the fourfold Cu-TiO_2_ coating investigated.

The blood tests taken did not show any signs of severe impairment of liver and kidney function within the investigated timeframe.

Drug-resistant microorganisms are responsible for a growing percentage of periprosthetic joint infections [31,32,33,34]. This problem is amongst other things associated with a large socio-economic cost, the treatment of an implant-associated infection caused by drug-resistant microorganims is 60% more expensive than the treatment for an implant-associated infection with non-drug-resistant microorganims [35]. The antibacterial Cu-TiO_2_ coating used in our study demonstrated a good efficacy against one of these problem microorganims, namely the Methicillin resistant *Staphylococcus aureus* (MRSA) class [28]. Thus the Cu-TiO_2_ coating investigated in our study prospectively represents a new method in the treatment of periprosthetic infections. It would be an alternative as well as a diversication of the currently used and established treatment by use of implanted antibiotic-carriers.

In order to provide a deeper understanding and to minimize possible biological hazard, further investigations are obviously needed before clinical application.

## Figures and Tables

**Table 1:** Mean value (m) and standard deviation (SD) of each measured parameter of group 1–3 and progression over 4 weeks.

	Units	Group	Preoperative Mean n ± SD	Week 1 Post-Operatively Mean n ± SD	Week 1 Post Revision Mean n ± SD	Week 2 Post Revision Mean n ± SD
Copper	(μg/dL)	group 1	75.98 ±14.86	197.21 ± 9.76	138.32 ± 73.30	126.2 ± 61.14
		group 2	71.61 ± 17.4	209.45 ± 105.2	178.06 ± 92.77	176.91 ± 92.03
		group 3	73.13 ± 8.3	233.51 ± 81.32	232.57 ± 102.58	153.03 ± 73.76
Ceruloplasmin	(mg/dL)	group 1	34.68 ± 9.68	87.84 ± 56.68	65.80 ± 49.20	52.55 ± 31.53
		group 2	26.03 ± 6.68	115.71 ± 67.12	100.92 ± 48.68	99.39 ± 58.08
		group 3	25.98 ± 5.27	138.07 ± 67.94	153.67 ± 81.37	69.53 ± 34.80
C-reactive Protein	(μg/mL)	group 1	5.2 ± 1.32	33.43 ± 28.53	11.82 ± 16.44	16.14 ± 19.85
		group 2	4.03 ± 1.07	48.49 ± 37.58	31.54 ± 21.63	31.21 ± 28.87
		group 3	3.89 ± 0.94	50.48 ± 27.73	36.46 ± 23.25	21.20 ± 21.24
Leucocytes	(1000/μL)	group 1	6.83 ± 1.32	8.37 ± 2.47	9.23 ± 3.79	7.74 ± 2.28
		group 2	6.31 ± 1.25	8.07 ± 1.15	10.91 ± 4.11	9.24 ± 3.04
		group 3	6.57 ± 1.55	8.37 ± 1.97	10.37 ± 3.43	7.16 ± 2.9
Glutamic-Pyruvate Transaminase (GPT)	[U/l]	group 1	32.57 ±15.82	38.12 ± 82.9	35.58 ± 10.82	32.25 ± 11.24
		group 2	38.14 ±11.44	72.59 ± 50.20	33.83 ± 29.73	29.73 ± 12.25
		group 3	40.25 ± 14.59	51.5 ± 25.42	42.50 ± 24.16	34.83 ± 15.58
Glutamic-Oxaloacetic Transaminase (GOT)	[U/l]	group 1	16.25 ± 4.41	31.38 ± 20.54	33.75 ± 22.2	31.38 ± 16.3
		group 2	21.76 ± 5.98	46.94 ± 30.65	27.33 ± 13.4	42.09 ± 36.89
		group 3	23.12 ± 4.87	42.12 ± 10.05	37.38 ± 10.76	36.00 ± 12.66
Gamma-Glutanyl-Transferase (GGT)	[U/l]	group 1	5.88 ± 1.82	6.88 ± 2.35	7.92 ± 2.23	8.38 ± 2.5
		group 2	6.14 ± 2.15	8.76 ± 4.12	9.17 ± 3.69	8.36 ± 1.22
		group 3	5.75 ± 1.38	6.88 ± 1.4	9.62 ± 6.27	8.17 ± 3.5
Creatinine	(mg/dL)	group 1	0.75 ± 0.12	0.83 ± 0.14	0.80 ± 0.18	0.91 ± 0.2
		group 2	1.00 ± 0.29	1.08 ± 0.25	0.84 ± 0.2	0.8 ± 0.15
		group 3	0.81 ± 0.23	0.83 ± 0.18	1.13 ± 0.66	0.81 ± 0.22
